# Redox Status and Telomere–Telomerase System Biomarkers in Patients with Acute Myocardial Infarction Using a Principal Component Analysis: Is There a Link?

**DOI:** 10.3390/ijms241814308

**Published:** 2023-09-20

**Authors:** Aleksandra Vukašinović, Aleksandra Klisic, Barbara Ostanek, Srdjan Kafedžić, Marija Zdravković, Ivan Ilić, Miron Sopić, Saša Hinić, Milica Stefanović, Nataša Bogavac-Stanojević, Janja Marc, Aleksandar N. Nešković, Jelena Kotur-Stevuljević

**Affiliations:** 1 Department of Medical Biochemistry, Faculty of Pharmacy, University of Belgrade, 11000 Belgrade, Serbia; alsavu@gmail.com (A.V.); miron.sopic@pharmacy.bg.ac.rs (M.S.); naca@pharmacy.bg.ac.rs (N.B.-S.); jkotur@pharmacy.bg.ac.rs (J.K.-S.); 2 Faculty of Medicine, University of Montenegro, 81000 Podgorica, Montenegro; 3 Center for Laboratory Diagnostics, Primary Health Care Center, 81000 Podgorica, Montenegro; 4 Department of Clinical Biochemistry, Faculty of Pharmacy, University of Ljubljana, 1000 Ljubljana, Slovenia; barbara.ostanek@ffa.uni-lj.si (B.O.); janja.marc@ffa.uni-lj.si (J.M.); 5 Department of Cardiology, Clinical Hospital Center Zemun, 11070 Belgrade, Serbia; skafedzic@yahoo.com (S.K.); ivan1ilic@yahoo.com (I.I.); milicastef89@gmail.com (M.S.); neskovic@hotmail.com (A.N.N.); 6 Faculty of Medicine, University of Belgrade, 11000 Belgrade, Serbia; dr.marija.zdravkovic@gmail.com; 7 Department of Cardiology, Clinical Hospital Center Bezanijska Kosa, 11070 Belgrade, Serbia; hinicsasa@yahoo.com

**Keywords:** atherosclerosis, acute myocardial infarction, redox status, telomere length, novel biomarkers

## Abstract

In the present study, we examined redox status parameters in arterial and venous blood samples, its potential to predict the prognosis of acute myocardial infarction (AMI) patients assessed through its impact on the comprehensive grading SYNTAX score, and its clinical accuracy. Potential connections between common blood biomarkers, biomarkers of redox status, leukocyte telomere length, and telomerase enzyme activity in the acute myocardial infarction burden were assessed using principal component analysis (PCA). This study included 92 patients with acute myocardial infarction. Significantly higher levels of advanced oxidation protein products (AOPP), superoxide anion (O_2_^•−^), ischemia-modified albumin (IMA), and significantly lower levels of total oxidant status (TOS) and total protein sulfhydryl (SH-) groups were found in arterial blood than in the peripheral venous blood samples, while biomarkers of the telomere–telomerase system did not show statistical significance in the two compared sample types (*p* = 0.834 and *p* = 0.419). To better understand the effect of the examined biomarkers in the AMI patients on SYNTAX score, those biomarkers were grouped using PCA, which merged them into the four the most contributing factors. The “cholesterol–protein factor” and “oxidative–telomere factor” were independent predictors of higher SYNTAX score (OR = 0.338, *p* = 0.008 and OR = 0.427, *p* = 0.035, respectively), while the ability to discriminate STEMI from non-STEMI patients had only the “oxidative–telomere factor” (AUC = 0.860, *p* = 0.008). The results show that traditional cardiovascular risk factors, i.e., high total cholesterol together with high total serum proteins and haemoglobin, are associated with severe disease progression in much the same way as a combination of redox biomarkers (pro-oxidant-antioxidant balance, total antioxidant status, IMA) and telomere length.

## 1. Introduction

Cardiovascular diseases (CVD) with acute coronary syndrome (ACS) still represent the leading cause of deaths worldwide [1]. About 17.9 million adults died from CVD in the world during 2019 [2], while in the last two years, the SARS-CoV-2 pandemic further increased this number due to limited regular medical check-ups. ACS includes a wide spectrum of events ranging from unstable angina pectoris to acute myocardial infarction (AMI) [3,4], where the most common underlying pathophysiological event is coronary atherosclerosis leading to artery occlusion [3]. Depending on the presence of ST-segment elevation and elevated cardiac biomarkers from blood (like cardiac Troponin I), AMI is differentiated into AMI with ST-segment elevation (STEMI: usually a consequence of complete and prolonged occlusion of a coronary artery blood vessel, followed by elevated cardiac biomarkers), and AMI without ST-segment elevation (non-STEMI: a consequence of a severe coronary artery narrowing, transient occlusion, or micro-embolization of thrombus and/or atheromatous material) [5,6].

According to European Society of Cardiology’s guidelines [7], the preferred strategy for STEMI patients is mechanical revascularization of the occluded artery through primary percutaneous coronary intervention (pPCI) within 12 h of the symptoms’ onset, preserving left ventricular systolic function, and reducing the onset of heart failure [7]. In order to estimate coronary artery disease (CAD) complexity, an important angiographic grading tool named the SYNTAX score has been developed. The SYNTAX score refers to the sum of the points assigned to each individual lesion identified in the coronary three with more than 50% diameter narrowing in vessels greater than 1.5 mm in diameter in the patient [8,9]. According to a meta-analysis by Bundhun et al., values of SYNTAX score above 17 are considered high, and those patients are prone to more severe complications following PCI, and worse outcome [9].

An imbalance between pro-oxidant production and antioxidant defence leads to the redox homeostasis disturbance, followed by overload of reactive oxygen species (ROS) and oxidative stress development [10]. Once it occurs, oxidative stress might also cause DNA damage, particularly at its ends, involving the telomere DNA, most probably via the formation of 8-oxo-7,8-dihydro-2′-deoxyguanine (8-oxoG) and single-strand breaks [11,12]. Likewise, oxidative stress might affect telomerase enzyme activity as well, which is crucial for telomere DNA prolonging and maintenance, causing its reactivation or inhibition [13,14], Although precise mechanisms are not completely understood, the presence of 8-oxoG and single-strand breaks in telomere DNA is thought to stimulate telomerase enzyme activity [13].

Regarding the samples used in the analysis, the most common one in clinical practise is peripheral venous blood, while arterial blood remains quiet unused. There are studies indicating that arterial and venous blood samples have comparable levels of common biochemical biomarkers or acid-base metabolites [15], but still there are no studies showing whether they have comparable levels of redox status biomarkers.

The first aim of our study was to evaluate the redox status biomarkers and parameters of the telomere–telomerase system (leukocyte telomere length and telomerase enzyme activity) in two sample types (arterial and peripheral venous blood) obtained at the same time point from AMI patients. The second aim was to define novel variables using a statistical tool: principal component analysis (PCA). The third aim was to evaluate the association of novel extracted factors with grading system which evaluates the complexity of coronary artery lesions and overall prognosis (SYNTAX score) in patients undergoing PCI. Lastly, the fourth aim was to evaluate the clinical accuracy of novel extracted variables in AMI patients in the study.

## 2. Results

Patients’ basic demographic characteristics and venous blood levels of analysed biomarkers are presented in Table 1.

Levels of the redox status biomarkers and parameters of the telomere–telomerase system were compared between venous and arterial blood samples of AMI patients (Table 2). Among pro-oxidant biomarkers or products of their activity, we found significantly higher levels of O_2_^•−^, AOPP, and IMA in arterial blood compared to the peripheral venous blood samples, while levels of TOS and SH-groups were lower in arterial than in peripheral venous blood samples. Parameters of telomere–telomerase system did not show statistically significant difference between two sample types. Additionally, we noticed positive correlation between left chamber ejection fraction rate and superoxide anion (ρ = 0.287; *p* = 0.077).

Abbreviations: AOPP, advanced oxidation protein products; TG, triglycerides; SH, sulfhydryl groups; PAB, pro-oxidant antioxidant balance; TAS, total antioxidant status; TOS, total oxidant status; O_2_^•−^, superoxide anion; SOD, superoxide dismutase, PON1, paraoxonase activity; IMA, ischemia modified albumin; MDA, malondialdehyde; LTL, leukocyte telomere length.

In order to reduce the number of initial variables, we combined them into a smaller number of factors using PCA, as summarised in Table 3. PCA was applied to the oxidative stress biomarkers, basic biochemical parameters (triglycerides (TG), total serum proteins, total cholesterol, haemoglobin, TnI, creatine kinase (CK activity) and body mass index (BMI)), and LTL and telomerase activity. The four extracted factors in peripheral venous blood explained 48.6% of the variance of all the evaluated variables. The first extracted factor included AOPP, TG and SH groups, and accounted for 17.1% of the total variance. It was entitled the “triglyceride–protein factor”. The second extracted factor explained 12.1% of the total variance and was composed of PAB, TAS, IMA and LTL, and was named the “oxidative–telomere factor”. The “cardiovascular disease biomarkers factor” was the third extracted factor that included Troponin I, CK activity and BMI, and explained 10.9% of the total variance. The “cholesterol–protein factor”, the fourth extracted factor, accounted for 8.5% of total variance and was characterised with the positive loadings of haemoglobin, total cholesterol, and total serum proteins.

PCA was also conducted for the oxidative stress and telomere–telomerase system parameters analysed in the arterial blood of the patients (Table 3). Three extracted parameters explained 65.9% of all the evaluated variance. The first one showed positive loadings of O_2_^•−^, PAB, TAS and IMA. It explained 43.3% of total variance and we named it the “oxidative factor”. The other two factors both accounted for around 11% of total variance, and were named the “arterial oxidative telomere factor” and the “oxidative telomerase factor”, respectively.

Abbreviations: AOPP, advanced oxidation protein products; TG, triglycerides; SH, sulfhydryl groups; PAB, pro-oxidant antioxidant balance; TAS, total antioxidant status; IMA, ischemia-modified albumin; LTL, leukocyte telomere length; CK, creatine kinase; BMI, body mass index; O_2_^•−^, superoxide anion; PON1, paraoxonase activity; TOS, total oxidant status; SOD, superoxide dismutase.

In order to evaluate if some of the new factors are associated with high values of the grading system evaluating CAD complexity (regarding the number of occluded coronary vessels or additional procedures needed) and the prognosis of STEMI patients undergoing pPCI (or SYNTAX score), a binary logistic regression analysis (enter selection) on both sample types (peripheral venous and arterial blood) was performed. Table 4 summarises the obtained results. The SYNTAX score was divided into tertiles, where the lowest tertile considered values lower than 10, and the higher tertile considered values over 17, as suggested by the Head research group [8]. The two best factors able to predict high SYNTAX score values in peripheral venous blood samples were “Oxidative telomere factor” (OR = 0.427; *p* = 0.035) and “Cholesterol-protein factor” (OR = 0.379; *p* = 0.008). Increased values of both factors in patients indicate that they are less prone to having high SYNTAX score values for 4.27% and 3.8%, respectively. On the other side, none of the new extracted factors in arterial blood samples adequately predicted the high SYNTAX score.

## 3. Discussion

Peripheral venous blood is a commonly used sample in clinical practice, while arterial blood samples are quite neglected, apart from in acid–base status and blood gases analysis [16,17]. Moreover, most research studies that includ AMI patients were performed on peripheral venous blood samples collected at various time points ranging from the moment of the acute event until recovery [18,19,20], or on blood vessels in in vivo or in vitro studies [21,22]. The experiments of Szasz and colleagues on healthy rat aorta and vena cava revealed a significant difference regarding ROS metabolism [23]. A higher ROS production followed by higher expression of major ROS-metabolizing enzymes (like xanthine oxidase, CuZn- superoxide-dismutase, and catalase) were confirmed in vena cava compared to aorta samples [23]. Along with that, we compared redox status parameters and parameters of telomere–telomerase system in peripheral venous and arterial blood samples of the AMI patients. We have found comparable levels for LTL and telomerase enzyme activity as well as most of the measured oxidative stress parameters, except IMA, O_2_^•−^ and AOPP, which were significantly higher in arterial blood compared to peripheral venous blood samples, indicating more serious oxidative stress condition in the arterial bloodstream. Along with this finding, we also measured significantly lower TOS and total SH-groups in arterial compared to venous samples (Table 2). Severe oxidative stress in the arterial blood is most probably caused by spontaneous reperfusion, which usually starts even before the pPCI procedure, as indicated by Börekçi and his research group [24]. Oxidative stress is involved in the many steps of atherosclerosis that precede AMI. It is already known that ROS in blood vessel walls play a crucial role in the pathogenesis of atherosclerosis via oxidized LDL formation. During the disease’s progression, oxidative stress is considered to participate in the vulnerability of the plaque’s fibrous cap, leading to its rupture, which is a hallmark of myocardial infarction. Moreover, it has been noticed that myeloperoxidase-derived reactive oxygen increased the release of tissue factor, leading to a thrombotic state. Therefore, increased oxidative stress in the pre-infarction state might be reduced through a spontaneous reperfusion by activating thrombus formation in the affected arteries and impairing endothelial function. On the other hand, thrombus formation might lead to a complete occlusion of arteries that consequently may increase oxidative stress in patients with STEMI, which is most probably reflected to the arterial blood as well [24]. The precise mechanism of different pro-oxidant regulation in arterial and venous blood remains unclear, since the studies reported up to now have opposing results. Some studies notices higher superoxide and hydrogen peroxide production in veins compared to arteries and higher NO production in arteries compared to veins [23]. The experiments of the Shrestha research group showed that venous endothelial cells are more sensitive to oxidant changes than arteries, since they have lower GSH:GSSG ratios. On the other side, arterial endothelial cells have higher levels of GSH compared to venous endothelial cells, so they appear to be more sensitive to changes in ROS [22]. Our results support this finding as arterial blood samples were collected at the admission, before the PCI procedure, where we found comparable levels of oxidative stress biomarkers, but interestingly, lower TOS levels and increased levels of its metabolites (like MDA) in arterial compared to venous blood samples. TOS is a measure of the complete pro-oxidant burden (a sum of H_2_O_2_ and lipid hydro-peroxides) in the blood, considering their complex and additive effects [25]. Since the radicals are unstable structures prone to degradation, an increased amount of degradation products could be detected in AMI patients. Indeed, our results sustain this theory, and we measured slightly higher values of MDA (degradation products of lipids) in the arterial blood samples compared to venous ones. Still, further studies are warranted to understand this complex mechanism.

Apart from the oxidative stress, in the AMI pathology, the role of telomere–telomerase system has been established, as well as its mutual interplay with oxidative stress [11,26]. Furthermore, the involvement of routine biochemical and hematological biomarkers (like lipids, C-reactive protein (CRP), white blood cell count, etc.) in AMI pathology and progress is already known [27], and they are usually interpreted or grouped based on their similarities or origin. Making a step further and using PCA analysis, the routine biomarkers in this study were combined (based on their variability, even if they do not have the same origin or role in the AMI pathophysiology) into the novel parameters. In this way, the obtained novel factors might be more comprehensive and might have a greater ability to reflect AMI patients’ pathophysiology or their outcome, which would be of clinical importance. The impact of newly extracted factors on AMI patients’ outcome was evaluated through their influence on the SYNTAX score. A high predicted SYNTAX score is of a great importance, since it represents an objective measure of CAD complexity and serves as a useful tool in terms of communicating the severity of disease and understanding its prognostic implications [28]. Moreover, studies also reported the SYNTAX score to be an effective reflection of atherosclerotic plaque severity [29,30]. In this study, combination of oxidative stress parameters and LTL in peripheral venous blood sample made the “Oxidative-telomere factor” (that included positive loadings of PAB, TAS, IMA and LTL), indicating that around 12% of variance in the SYNTAX score is related to the changes in those four parameters (Table 3 and Table 4). Van Belle and collaborators have already noticed independent increased values of IMA in AMI patients [31], and suggested them to be strong and independent predictors of 1-year cardiac outcome in CVD patients. Moreover, IMA levels measured in first 24 h from the admission showed the ability to identify patients who require a different medical approach, such as intra-aortic balloon pump counter-pulsation or PCI [30]. Oppositely, Panjwani and colleagues suggested that IMA could not be used as an independent parameter for the identification of AMI, since outcome may depend on the concentration of other factors (like serum albumin) [32]. Likewise, PAB is considered a comprehensive biomarker measuring concurrently plasma pro-oxidant activity and antioxidant capacity, and it has already been recommended as a convenient parameter for oxidative stress evaluation in patients with STEMI by various groups [33,34,35]; higher values of PAB point to the increased levels of oxidative stress. On the other side, decreased TAS values were noticed in several CAD studies [36,37], but its predictive ability has not been reported until now. Different studies reported short LTL as an independent predictor of short-term major adverse cardiovascular events (MACEs) and long-term outcomes in CAD patients. There is an inverse association of LTL with the risk of coronary heart disease which is independent of conventional vascular risk factors [38]. In addition, oxidative stress is considered a possible contributor to LTL attrition in individuals at high risk of CAD. The exact mechanism of telomere shortening induced by the oxidative stress burden is not completely revealed, but most probably, ROS released under the conditions of oxidative stress influence guanine structure by creating its modified bases (8-oxo-7,8-dihydro-2′-deoxyguanine and causing) and creating single-strand breaks in telomere DNA that lead to genome instability and also to telomere attrition [12]. Therefore, to the best of our knowledge, this unique combination of peripheral oxidative-stress markers and LTL, which is able to identify higher SYNTAX score values and consequently indicate more complex states and severe prognoses of AMI patients undergoing pPCI, has not been reported up to now.

The second extracted factor consisting of hemoglobin, total cholesterol, and total serum proteins, termed the “cholesterol–protein factor”, had a similar predictive ability regarding high SYNTAX scores. Low hemoglobin concentrations are usually linked to anemia, which in the ACS setting might worsen myocardial ischemia, since there is already insufficient oxygen supply to the myocardium; however, data relating anemia to clinical outcomes in ACS are still limited. The Sabatine research group [39] reported significant and independent associations between low hemoglobin concentrations and adverse cardiovascular outcomes in a broad cohort of ACS patients. There was a progressive increase in cardiovascular mortality and heart failure among patients with STEMI, as the baseline hemoglobin dropped below 14 g/dL (140 g/L), indicating that anemia could be a powerful and independent predictor of MACEs in patients across the spectrum of ACS [39]. Moreover, Feng and collaborators looked at hemoglobin’s association with age, albumin, and creatinine in patients with AMI [40]. In addition, there are no data that point to a direct connection between hemoglobin concentration and SYNTAX score. On the other side, lipoprotein subclasses are a well-characterized risk factor for AMI [41]. A standard lipid panel containing high levels of TG, high non-high-density lipoprotein cholesterol (non-HDL-C) and low HDL-cholesterol (HDL-C) is correlated to a high risk of CVD. More comprehensive lipid testing identified an increased levels of small dense LDL particles and remnant lipoproteins as particularly atherogenic ones [42]. Moreover, Xu’s research group reported the association of Lp(a) level with SYNTAX score, which was maintained for the group with LDL-C values over 100 mg/dL (2.586 mmol/L) [42].

Regarding total protein levels, there are no individual data, since only the correlation of hsCRP and albumin to the SYNTAX score was reported. The precise connection between these parameters has not been described, but multiple mechanisms might be involved. Inflammation plays an important role in all stages of atherosclerosis, meaning that a decreased albumin level and increased CRP level are associated with the chronic nature of the disease [43]. High CRP has may be involved in many processes such as uptake of LDL-C by macrophages and its turning into foam cells, while a decreased albumin level is associated with increased blood viscosity, impaired endothelial function, increased platelet activation and aggregation, and increased synthesis of platelet-derived coronary artery narrowing mediator (like prostaglandin D2) [43]. All these processes contribute to the progression of atherosclerosis, which might be reflected by the SYNTAX score, since Karabağ and collaborators reported the hsCRP/albumin ratio to be a tightly associated indicator of CAD complexity and severity and an independent predictor of an intermediate-to-high SYNTAX score [43]. Still, further research is needed to understand this intriguing connection.

## 4. Materials and Methods

### 4.1. Patients

The subjects in the study were patients with AMI (N = 92) admitted to the intensive care units of Clinical Hospital Center Zemun, Belgrade, Serbia, and Clinical Hospital Center “Bezanijska kosa”, Belgrade, Serbia. The inclusion criteria were patients of both genders aged between 18 and 80 years, with infarction pain present for a maximum of 12 h and characteristic clinical symptoms. Myocardial necrosis was assessed using cardiac troponin I (cTnI) levels, while myocardial infarction was confirmed using coronary angiography. The study group included STEMI patients, identified after the appropriate diagnostic procedure in the intensive care unit.

All participants were informed about the purpose and the aim of the study, and signed informed consent before they were included in the research. The Ethical Committees of Clinical Hospital Center Zemun (No. 325/1, from 24 September 2015) and Clinical Hospital Center “Bezanijska kosa” (No. 4705/4, from 31 May 2016) approved the study protocol.

### 4.2. Sample Collection and Measurement

Blood samples were obtained from patients upon the admission to the Emergency units, before pPCI, as follows: one sample of peripheral venous blood (cubital vein) and one sample of arterial blood (iliac artery).

Peripheral venous blood samples [i.e., serum, plasma and whole blood samples (i.e., for telomerase enzyme and DNA isolation)] were drawn into collection tubes containing serum separator gel for serum samples and EDTA as an anticoagulant for plasma and whole blood samples. Serum and plasma samples were separated from blood cells after centrifugation, while the whole blood samples were immediately processed for telomerase enzyme and genomic DNA extraction. Oxidative stress status parameters, antioxidant status markers, and basic biochemical parameters were determined in the serum or plasma samples of patients.

Redox status and routine biochemical parameters were measured on an ILAB 600 analyser (Instrumentation Laboratory, Milan, Italy). For the measurement of biochemical parameters, routine commercial methods were used (total cholesterol, triglycerides, total blood proteins, creatine kinase activity, and haemoglobin), and were implemented with an ILAB 600 analyser (Instrumentation Laboratory, Milan, Italy). Redox status parameters [i.e., paraoxonase activity (PON1), advanced oxidation protein products (AOPP), superoxide anion (O_2_^•−^), ischemia-modified albumin (IMA), total oxidant status (TOS), total antioxidant status (TAS), pro-oxidant-antioxidant balance (PAB), malondialdehyde (MDA), and total protein sulfhydryl (SH-) groups] were measured using methods validated in our laboratory [44]. Levels of troponin I were determined using a commercial Access Immunoassay system (UniCel DxI 600 Access Immunoassay System, Beckman Coulter Inc., Brea, CA, USA). The telomerase enzyme activity was measured using a modified Real-Time Telomeric Repeat Amplification Protocol (RTq-TRAP), as described previously [45]. Leukocyte telomere length (LTL) was determined with modified qPCR and calculated as the T/S ratio [46].

### 4.3. Statistical Analysis

Normality distribution for all variables was assessed using the Kolmogorov–Smirnov test. Data are presented as mean ± standard deviation for reaans with 25th and 75th percentile value for variables with non-normal distribution. Parameters with normal distribution were analysed using Student’s *t*-test. Asymmetrically distributed variables were assessed using the Mann–Whitney U test, and frequencies with Chi-square tests using contingency tables. A PCA was further conducted in order to reduce the number of examined variables into a smaller number of factors, and a varimax-normalized rotation was used. Normally distributed variables and variables with skewed distribution after logarithmic transformation data were processed. An eigenvalue > 1 was used for the extracted factors, while variables with factor loadings > 0.5 were used for the interpretation of factors. As independent variables in the subsequent logistic regression analysis, the scores calculated for factors with eigenvalues > 1 were included. To evaluate the potential impact of newly formed factors in PCA analysis on SYNTAX score in AMI patients, binary logistic regression analysis (enter selection) was used. All statistical analyses were performed using PASW^®^ Statistic v.18 (Chicago, IL, USA) software. A *p* value < 0.05 was considered statistically significant.

## 5. Conclusions

In the present study, an important association between traditional risk factors (like high total cholesterol, total plasma proteins and haemoglobin), redox status parameters (PAB, TAS, IMA), LTL, and the severity of AMI patients’ states, observed through SYNTAX score, has been established. This simple approach could be a very useful tool for the development of more precise and comprehensive biomarkers in the future, which as a potential part of personalised medicine could be supplemented by patients’ clinical or anamnestic data.

## Figures and Tables

**Table 1:** Basic demographic characteristics and levels of examined biomarkers in the study participants.

Parameter	AMI Patients
N	92
Age, years ^#^	60.8 ± 11.72
Body mass index, kg/m^2^	25.7 (23.6–28.7)
Syntax score, points	13 (8–19)
High blood pressure, %	29.7
Smokers, %	42.3
Dyslipidaemia, %	49.3
Glucose intolerance, %	11.8
Statins, %	17.2
Coronary vessels with atherosclerotic occlusion, number	1–5
Implanted stents, number	1–5
Left chamber ejection fraction rate, %	42.1
BMI, kg/m^2^	25.7 (23.6–28.7)
Triglycerides, mmol/L	1.75 (1.20–2.39)
Total cholesterol, mmol/L	5.58 (4.66–6.42)
Total blood proteins, g/L	69.5 (65.0–75.0)
Troponin I, mg/L	0.41 (0.07–2.93)
Creatine kinase activity, IU/L	204 (100–487)
Haemoglobin, g/L ^#^	144 ± 16.2

The results are presented as medians with 25th and 75th percentile values, mean value ± standard deviation for normally distributed variables (#), and as percentages for frequencies.

**Table 2:** Circulating levels of examined biomarkers in peripheral and arterial blood samples in the study participants.

Parameter	AMI Patients		*p*
	Peripheral Blood Sample	Arterial Blood Sample	
AOPP, μmol/L	25.6 (14.7–35.6)	51.9 (37.8–76.2)	<0.001
Total SH groups, mmol/L	0.443 (0.325–0.561)	0.344 (0.255–0.382)	<0.001
PAB, U/L	117 (102–133)	106 (87–152)	0.388
TAS, μmol/L	910 (771–1138)	916 (481–1415)	0.496
TOS, μmol/L	20.4 (8.0–27.9)	8.7 (5.1–19.2)	0.002
O_2_^•−^, μmol/L NBT/min/L	56 (38–77)	160 (48–255)	<0.001
SOD, U/L	141 (124–187)	155 (109–203)	0.695
PON1, U/L	284 (172–474)	275 (166–618)	0.609
IMA, absorbance units	0.296 (0.078–0.405)	0.486 (0.406–0.593)	<0.001
MDA, μmol/L	3.26 (2.44–6.22)	3.96 (3.43–4.63)	0.743
Leukocyte telomere length, T/S ratio	1.117 (0.928–1.343)	1.144 (0.868–1.589)	0.834
Telomerase activity, log activity	0.375 (0.350–0.396)	0.359 (0.345–0.387)	0.419

Results are presented as medians with 25th and 75th percentile value and were analysed using a Mann–Whitney test.

**Table 3:** Extracted factors by PCA.

Sample Type	Factors	Included Variables with Loadings	Factor Variability—Single (%)	Factor Variability—Cumulative (%)
Peripheral Blood *	Triglyceride–protein factor	AOPP (0.748) TG (0.733) SH-groups (0.627)	17.1	48.6
	Oxidative–telomere factor	PAB (−0.734) TAS (0.669) IMA (0.624) LTL (0.504)	12.1	
	Cardiovascular disease biomarker factor	Troponin I (0.891) CK-activity (0.864) BMI (−0.585)	10.9	
	Cholesterol–protein factor	Haemoglobin (0.663) Total cholesterol (0.640) Total serum proteins (0.592)	8.5	
Arterial Blood **	Oxidative factor	O_2_^•−^ (0.829) PAB (0.797) TAS (0.731) IMA (0.563)	43.3	65.9
	Arterial oxidative–telomere factor	LTL (0.790) PON1 (0.766) TOS (0.629)	11.4	
	Oxidative–telomerase factor	Telomerase activity (−0.855) SOD (−0.501) TOS (0.545)	11.2	

* Kaiser–Meyer–Olkin measure of sampling adequacy for peripheral serum samples = 0.613; ** Kaiser–Meyer–Olkin measure of sampling adequacy for arterial serum samples = 0.716.

**Table 4:** Binary logistic regression analysis of predictors for SYNTAX score high values (>17) in peripheral blood samples.

Sample Type	Predictors	OR	95th CI	*p*
Peripheral Blood	Triglyceride–protein factor	2.063	0.998–4.266	0.051
	Oxidative telomere factor	0.427	0.194–0.943	0.035
	Cardiovascular disease biomarker factor	0.876	0.506–1.518	0.637
	Cholesterol–protein factor	0.379	0.184–0.777	0.008
Arterial Blood	Oxidative factor	0.481	0.208–1.116	0.088
	Arterial oxidative telomere factor	1.634	0.727–3.671	0.235
	Oxidative telomerase factor	1.086	0.455–2.590	0.853

OR = odds ratio; CI = confidence interval.

## Data Availability

The data will be available upon reasonable request (contact aleksandranklisic@gmail.com).
